# Loss of inducible nitric oxide synthase promotes Kras/Pten-driven lung tumorigenesis

**DOI:** 10.3389/fcell.2026.1630208

**Published:** 2026-02-27

**Authors:** Zahra Kabiri, Hamed Zaribafzadeh, Sara Raji, John M. Carney, Christopher M. Counter

**Affiliations:** 1 Department of Pharmacology & Cancer Biology, Duke University Medical Center, Durham, NC, United States; 2 Department of Surgery, Duke University Medical Center, Durham, NC, United States; 3 Department of Pathology, Duke University Medical Center, Durham, NC, United States

**Keywords:** iNOS, macrophage, NSCLC, nitric oxide, nitric oxide synthase, non-small cell lung cancer, PTEN, RAS

## Abstract

The inducible *N*itric *O*xide *S*ynthase (iNOS) enzyme has been implicated in both pro- and anti-tumorigenic processes, depending on the cancer context. In oncogenic Kras-driven mouse models of lung adenocarcinoma, the loss of iNOS reduces tumorigenesis. To explore the additional loss of the tumor suppressor Pten in this setting, we compared lung tumorigenesis in mice induced by activation of oncogenic Kras in conjunction with inactivation of Pten in the absence and presence of iNOS. We report that the loss of iNOS did not affect the number or type of lung lesions compared to control iNOS wild-type mice, but was associated with shortened overall survival that was accompanired by increased tumor burden and intratumoral macrophage infiltration. These findings suggest that the antineoplastic effect of iNOS deficiency in Kras-driven lung tumorigenesis is reversed upon the loss of Pten. Thus, even within the identical cancer model, the loss of iNOS can have opposite effects depending on the genetic context.

## Introduction

Lung cancer remains the leading cause of cancer-related mortality worldwide to date (Siegel et al., 2025). *N*on-*S*mall *Cell L*ung *C*ancer (NSCLC) accounts for approximately 85% of all cases, with *LU*ng *AD*enocarcinoma (LUAD) being the most prevalent histological subtype, representing nearly half of NSCLC diagnoses (Lin et al., 2024). While patients with early-stage LUAD may benefit from surgical resection, those with advanced or unresectable disease require systemic therapy (Hua et al., 2023). Recent advances in molecular oncology have led to the development of targeted therapies against oncogenic drivers such as EGFR (Zhou et al., 2011) and KRAS (Skoulidis et al., 2021), in addition to immunotherapeutic approaches targeting immune checkpoints (Paz-Ares et al., 2018). Despite these therapeutic advancements, the 5-year overall survival rate for LUAD still remains below 20%, largely due to the emergence of drug resistance (Malkoski et al., 2014). Thus, there is a pressing need for more effective and durable treatment strategies.

*N*itric *O*xide (NO) is a multifunctional molecule that serves as a key signaling mediator for a wide range of physiological processes, including vasodilation (Tousoulis et al., 2012), immune regulation, neural communication, programmed cell death, reproduction, gene transcription, mRNA translation, and protein post-translational modifications (Lim et al., 2008; Andrabi et al., 2023). In the context of cancer, NO exhibits a dual role, functioning as both a tumor promoter and a tumor suppressor depending on its concentration and source (Vannini et al., 2015). In more detail, NO has been shown to modulate the tumor microenvironment, contributing to tumor progression, tumor invasion, angiogenesis, and drug resistance in different cancers such as NSCLC (Puhakka et al., 2003; Pershing et al., 2016), pancreatic cancer (Fujita et al., 2019), and glioblastoma (Kruglyakov et al., 2023). Conversely, NO also displays tumor-suppressive effects, such as promoting apoptosis by inhibiting of autophagy in hepatocellular carcinoma cells (Zhang et al., 2019), reducing motility and enhancing adhesion in human breast cancer cells (Lahiri and Martin, 2009), and suppressing tumorigenesis through inducing apoptosis in the thymus of p53-deficient mice (Hussain et al., 2004).

*N*itric *O*xide *S*ynthase (NOS) catalyzes the conversion of L-arginine to L-citrulline and NO. There are three NOS isoforms: neuronal NOS (nNOS or NOS1), inducible NOS (iNOS or NOS2), and endothelial NOS (eNOS or NOS3). Each isoform is encoded by a distinct gene and expressed in different cell types with specific regulatory mechanism (Salimian Rizi et al., 2017). iNOS is typically induced to produce high levels of NO in response to inflammatory stimuli such as tumor necrosis factor-α, oxidative stress, and interferon-γ (Khan et al., 2020).

iNOS has been found to have both pro- and anti-tumor effects in lung cancer. In terms of the former, increased iNOS expression has been reported in tumor tissues but not adjacent normal lung tissue (Speranza et al., 2007). Moreover, circulating tumor cells from LUAD patients with bone metastases exhibit elevated iNOS protein expression, suggesting that iNOS may serve as a potential biomarker for metastatic progression in lung cancer (Zhang et al., 2014). In terms of the latter, elevated iNOS expression has been detected in cancer-associated fibroblasts, *T*umor-*I*nfiltrating *l*ymphocytes (TILs), and the tumor themselves in human NSCLC. Notably, the elevated iNOS expression in TILs was associated with improved patient survival, suggesting a role for NO-mediated immune activation in NSCLC (Giatromanolaki et al., 2020). iNOS expression was also found to be higher in lower-grade NSCLC, with high NOS expression correlating with improved survival (Puhakka et al., 2003). Thus, the effect of iNOS in lung cancer is complex and context-dependent.

Given that tumor suppressor loss is a major determinant of tumor cell state, immune interactions, and signaling network rewiring during LUAD progression, we hypothesized that inactivation of tumor suppressors may be a key factor governing the context dependent function of iNOS in lung cancer. In particular, loss of tumor suppressors such as PTEN are known to profoundly alter cellular metabolism, inflammatory signaling, and tumor immune crosstalk (Garcia et al., 2014; Liu Y. et al., 2024; Paredes et al., 2025; Zhao et al., 2025). These processes directly intersect with nitric oxide biology and provide a mechanistic framework through which iNOS function could be reprogrammed from tumor suppressive to tumor promoting, or *vice versa* (Ding et al., 2021; Kieler et al., 2021; You et al., 2024).

Genetically Engineered Mouse Models (GEMM) provide the opportunity to genetically interrogate signaling pathways in lung cancer. In this regard, inhalation of *Ad*enovirus encoding *Cre* recombinase (Ad-Cre) to recombine and activate the Cre-inducible (LSL) oncogenic (G12D) *Kras* allele (*Kras* ^*LSL-G12D*^) specifically in the lungs of mice generates lung adenomas that progress at a low frequency to LUAD (Jackson et al., 2001). Progression to LUAD is accelerated by simultaneously Cre-mediated inactivation of both floxed *Pten* tumor suppressor alleles (*Pten* ^*fl/fl*^) and activation of oncogenic (G12D) *Kras* allele (*Kras* ^*LSL-G12D/+*^) via a *C*lara *C*ell-*SP*ecific *Cre* (CCSP-Cre) recombinase (Iwanaga et al., 2008), highlighting the interplay of oncogenes and tumor suppressors in lung cancer progression. Of note, it has been reported that a homozygous null *iNOS* (*iNOS* ^*−/−*^) genotype reduces the number of lung lesions induced by activating the *Kras* ^*LSL-G12D*^ allele in the lungs of mice by Ad-Cre inhalation and extended survival (Okayama et al., 2013). Given that iNOS effects are context dependent, we evaluated the effect iNOS loss in this latter model when Pten was inactivated. Here we demonstrate that while the number and type of pulmonary lesions arising upon recombination and activating the *Kras* ^*LSL-G12D*^ allele by intranasal administration of adenovirus Cre was not changed when both alleles of *Pten* ^*fl*^ were recombined and inactivated, this did lead to an increase in tumor burden and reduced lifespan. These data support Pten loss reversing the anti-tumorigenic effect of iNOS loss on oncogenic-Kras-driven murine LUAD.

## Materials and methods

### Mice

*iNOS* ^−/−^ (Laubach et al., 1995), *Kras* ^*LSL-G12D/+*^ (Jackson et al., 2001), and *Pten* ^*fl/fl*^ (Lesche et al., 2002) mice obtained from Jackson laboratory (strains: 002609, 008179, and 006440, respectively) were used to generate *Kras* ^*LSL-G12D/+*^; *Pten* ^*fl/fl*^; *iNOS* ^−/−^ versus *Kras* ^*LSL-G12D/+*^;*Pten* ^*fl/fl*^; *iNOS* ^*+/+*^ littermates. At 2 to 3 months of age, these mice were intranasally administered 6 × 10^10^ pfu/mL Ad-Cre (University of Iowa) as previously described (Tousoulis et al., 2012). Mice were monitored twice weekly for changes in body weight and signs of morbidity. Mice were humanely euthanized at 60 days post-inhalation (tumor analysis study) or either upon reaching a moribundity endpoint or 100 days post-inhalation (survival study). Lungs were removed at necropsy and analyzed for the presence of tumors. All mice were housed under aseptic conditions in DLAR facility and cared according to IACUC guidelines. All procedures were approved by Duke University IACUC and conducted in compliance with NIH animal care regulations.

### Histology and scoring

Lungs were collected at necropsy, embedded in paraffin, cut into 5 μm sections, stained with hematoxylin and eosin (H&E), and scanned for histological analysis (The GT-450 Aperio Scanner, Duke University).

Immunohistochemical staining was performed using the automated Roche Discovery Ultra system under heat-induced epitope retrieval with EDTA buffer (pH 8.0) for 56 min at 99 °C. Sections were incubated with anti-CD68 antibody (Abcam, catalog no. ab125212), as previously described (Kabiri et al., 2018).

All histological slides were decoded, and a board-certified pathologist conducted a blinded assessment of all slides. To ensure consistency, lungs were serially sectioned, and the section containing the largest lung area was H&E-stained and analyzed (one section per mouse). The total number of lesions per lung was recorded and classified into three groups: fewer than 30 lesions, 30–60 lesions, and more than 60 lesions per lung. Tumor burden was assessed by estimating the percentage of lung area involved by lesions and categorized into three groups: 0%–25%, 26%–50%, and 51%–75% involvement.

Macrophage scoring intensity was assessed in a relative manner. Two distinct staining patterns were evaluated: peritumoral and intratumoral CD68 staining. All samples exhibited some degree of macrophage infiltration, with a baseline “salt-and-pepper” appearance observed across specimens. Overall CD68 staining was scored as mild (score 1), moderate (score 3), or marked (score 5) intensity. Peritumoral macrophages typically formed a pattern “wrapping around” tumor nodules rather than infiltrating tumor cell aggregates and were scored as mild (score 1), moderate (score 3), or marked (score 5). Intratumoral CD68 staining was characterized by either large aggregates of CD68-positive cells or patchy clusters with fewer cells and was scored as minimal (score 0), minimal to mild (score 0.5), mild (score 1), mild to moderate (score 2), or moderate (score 3) intensity.

### Sample size justification

Two groups of mice (n = 8 and n = 9 for tumor initiation study at 60-day endpoint and n = 7 per group in survival study) were studied. The chosen sample size is consistent with previous publication in similar *Kras* mouse models (Lampson et al., 2012) and represents a balance between obtaining robust data and adhering to the principle of reduction in animal research.

### Statistical analysis

Statistical comparisons of lesion counts and percentage of tumor-involved lung area between *Kras* ^*LSL-G12D/+*^; *Pten* ^*fl/fl*^; *iNOS* ^*−/−*^ versus *iNOS* ^*+/+*^ cohorts were performed using the Mann–Whitney U test. Longitudinal body weight differences were analyzed using two-way ANOVA. Kaplan–Meier survival curves were generated for each group, and statistical differences in survival were assessed using the log-rank test. All analyses were conducted using Prism 10.0 software (GraphPad Software, LLC, California, United States). A p-value of <0.05 was considered statistically significant.

## Results

**FIGURE 1:**
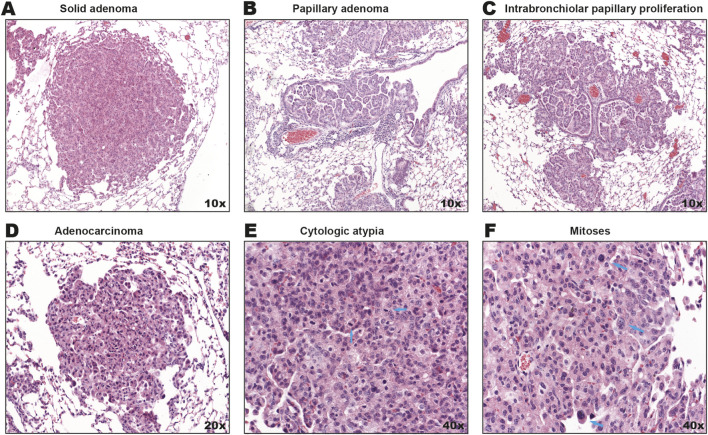
Inhaled Ad-Cre induces lung tumorigenesis in a *Kras* ^*G12D/+*^; *Pten* ^*fl/fl*^ background. **(A)** Representative example of a solid-type adenoma, **(B)** papilla adenoma with bland epithelial cells with no nuclear atypia or pleomorphism, **(C)** Solid and papillary type adenomas, foci of intrabronchiolar papillary proliferations. Adenocarinomas were also observed, as evidence of non-small cell lung carcinoma tumors with **(D)** glandular differentiation, **(E)** patchy foci of cytologic atypia (blue points) and, **(F)** foci of increased mitoses (blue points).

As noted above, activation of *Kras* ^*LSL-G12D*^ via inhalation of Ad-Cre generates lung adenomas in mice that progress at a low frequency to LUAD (Jackson et al., 2001), and that this progression is accelerated by simultaneous deletion of the tumor suppressor Pten via CCSP-Cre expression (Iwanaga et al., 2008). To address the effect of iNOS loss in the more aggressive setting of oncogenic Kras and Pten loss, we first tested whether this accelerated progression of LUAD is also observed via recombination by intranasal administered Ad-Cre. To this end, three *Kras* ^*LSL-G12D/+*^; *Pten* ^*fl/fl*^ mice were administered Ad-Cre via intranasal inhalation at 8–12 weeks of age, as we previously performed (Pershing et al., 2016). 60 days later when tumors should be prevalent (Iwanaga et al., 2008), all mice were humanely euthanized and their lungs removed during necropsy, section, stained by H&E, and analyzed by a pathologist. Similar to recombination via CCSP-Cre expression, we observed a range of lung tumor histologies. These included solid adenomas (Figure 1A), papillary adenomas (Figure 1B), intrabronchiolar papillary proliferations (Figure 1C), mixed solid-papillary lesions with complex architecture (Figure 1D), pronounced cytologic atypia (Figure 1E), and frequent mitotic figures (Figure 1F), all characteristic of invasive adenocarcinoma. These results confirm LUAD can be induced by Ad-Cre administration in this inducible mouse model, providing a valuable platform for studying tumor initiation, progression and therapeutic interventions without the extra effort required to cross in a Cre driver.

**FIGURE 2:**
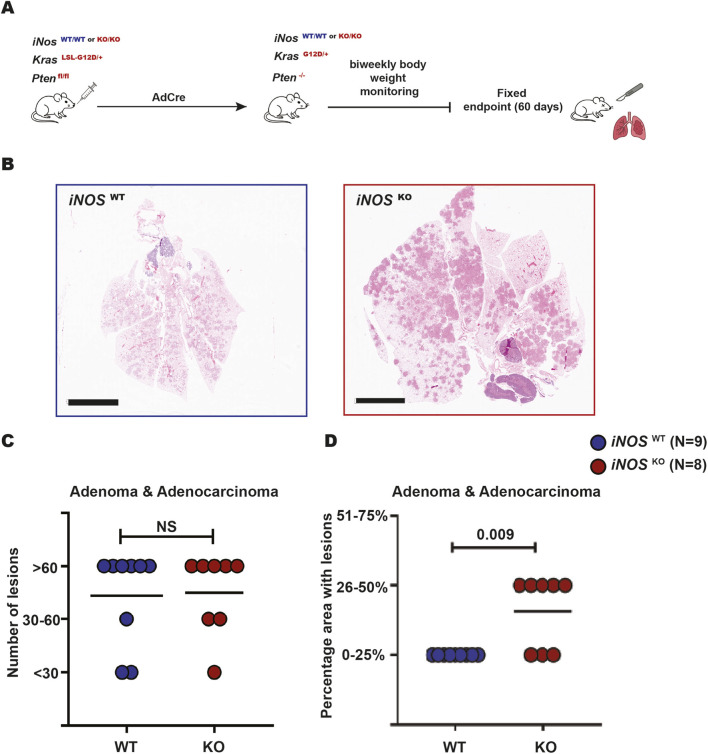
iNOS KO mice exhibit larger lung lesions. **(A)** Schematic of the experimental design used to assess the role of iNOS in tumor development. **(B)** Representative H&E-stained lung sections from WT (left) and KO (right) mice at 60 days endpoint. **(C)** Number of lesions (less than 30 [<30], 30 to 60 lesions [30–60], and more than 60 lesions [>60] and **(D)** percentage of lung area (categorized as 0%–25%, 26%–50%, and 51%–75%) occupied by adenoma or adenocarcinoma in iNOS WT (N = 9) versus KO (N = 8) mice. Mann–Whitney U test was used to compare number of lesions and percentage area with lesions between mice groups. NS = not significant.

To investigate the role of iNOS in lung tumor development in this GEMM of LUAD, *Kras* ^*LSL-G12D/+*^; *Pten* ^*fl/fl*^ mice were crossed with *iNOS* ^*-, -*^ mice. After several generations, we obtained *Kras* ^*LSL-G12D/+*^;*Pten* ^*fl/fl*^; *iNOS* ^+/−^ offspring, which were intercrossed to generate eight *Kras* ^*LSL-G12D/+*^; *Pten* ^*fl/fl*^; *iNOS* ^−/−^ (termed iNOS KO for *K*nock *O*ut) and nine control *Kras* ^*LSL-G12D/+*^; *Pten* ^*fl/fl*^ *iNOS* ^+/+^ (termed iNOS WT for *W*ild-*T*ype) littermates to account for background strain variability. At 8–12 weeks of age these mice received intranasal Ad-Cre. 60 days all mice were humanely euthanized and their lungs removed and analyzed as above (Figure 2A). Lungs from iNOS KO mice appeared visibly larger, likely reflecting more extensive tumor burden (Figure 2B). To more accurately interrogate this observation, a total of seventeen sections from both genotypes were scored in a blinded fashion by a pathologist for the types and number of lung lesions, and percentage of lung involvement.

In terms of tumor number, the number of lesions were partitioned into three groups: <30 lesions, 30–60 lesions, >60 lesions per section per mouse. In the control iNOS WT group, two mice had fewer than 30 lesions, one had 30–60 lesions, and six had more than 60 lesions. In the iNOS KO group, one mouse had fewer than 30 lesions, two had 30–60 lesions, and five had more than 60 lesions. Statistical analysis revealed no significant difference in the total number of lung lesions between these two groups (P > 0.9; Figure 2C), indicating that iNOS does not significantly influence early tumorigenesis in the absence of Pten. Interestingly, invasive adenocarcinoma was detected in four of nine mice in the iNOS WT group and in four of eight mice in the iNOS KO group, suggesting comparable frequencies of adenocarcinoma development across both genotypes.

In terms of tumor burden, the percentage of lung area occupied by tumor lesions relative to total lung area was partitioned into three groups: <25%, 26%–50%, and 51%–75% involvement. In the control iNOS WT group, all nine exhibited <25% tumor involvement. In the iNOS KO group, three mice exhibited <25% involvement and five had 26%–50% lung involvement, which was a statistically significant increase in tumor burden compared to the WT control group (P = 0.009; Figure 2D). Collectively, the increased lung size and higher percentage of tumor-involved lung area in iNOS KO mice at the 60-day endpoint suggest that iNOS deficiency enhances tumor burden in this genetic background.

**FIGURE 3:**
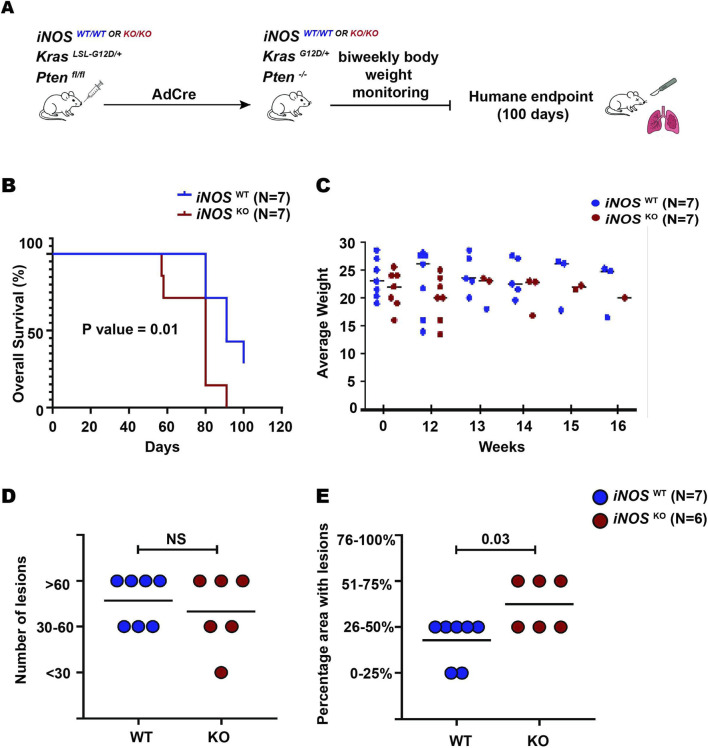
iNOS KO mice exhibit reduced overall survival and higher tumor burden. **(A)** Schematic of the experimental design used to assess the role of iNOS in tumor development. **(B)** Kaplan–Meier survival analysis (WT mice [N = 7], KO mice [N = 7]), **(C)** body weight (0 = AdCre infection, WT mice [N = 7], KO mice [N = 7]), longitudinal body weight differences were analyzed using two-way ANOVA. **(D)** Percentage of lung area (categorized as 0%–25%, 26%–50%, and 51%–75%) occupied by adenoma or adenocarcinoma and **(E)** number of lesions (less than 30 [<30], between 30 to 60 [30–60], and more than 60 [>60]) in iNOS WT (N = 7) versus KO mice (N = 6). Mann–Whitney U test was used to compare number of lesions and percentage area with lesions between mice groups. NS = not significant.

To evaluate whether iNOS deficiency affects overall survival in this model, we generated another two cohorts of littermates consisting of seven iNOS WT and seven iNOS KO mice using the same breeding strategy described above to control for genetic background. At 8–12 weeks of age, these mice received intranasal Ad-Cre, but in this case were humanely euthanized upon reaching a moribundity endpoint, or 100 days later (Figure 3A). Kaplan–Meier survival analysis demonstrated a significantly reduced median survival in the iNOS KO group compared to WT controls (median survival: 80 vs. 91 days; hazard ratio: 2.84; 95% confidence interval: 0.85–9.46; P = 0.01; Figure 3B). Notably, two iNOS WT mice survived to the 100-day endpoint without exhibiting any clinical signs of tumor burden or morbidity. Longitudinal monitoring of body weight revealed no significant differences between the two groups (Figure 3C). Thus, iNOS deficiency reduces survival in this genetic model of LUAD.

To assess tumor burden at endpoint, lung tissue removed at the time of necropsy was analyzed as above. In the iNOS WT group, two mice exhibited <25% lung involvement, while five showed 26%–50% involvement. In iNOS KO group, three mice had 26%–50% involvement and three more mice exhibited >50% tumor involvement, indicating a higher tumor burden in the iNOS KO group (Figure 3D). Despite this difference in tumor burden, the number of lung lesions did not significantly differ between the two cohorts (Figure 3E). Adenocarcinoma was observed in two of the six iNOS KO mice, but not in any of the iNOS WT mice. This increased tumor burden coincided with reduced survival, supporting iNOS loss promoting tumor progression, rather than tumor initiation, contributing to poorer survival outcomes.

**FIGURE 4:**
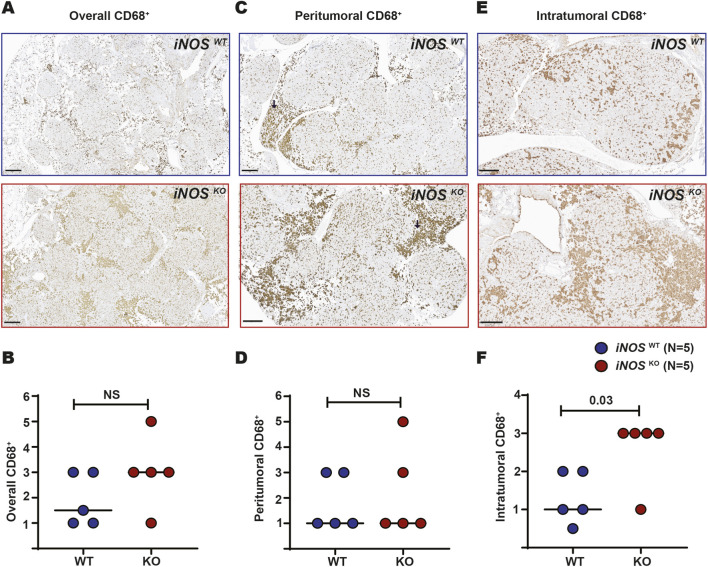
iNOS KO mice exhibit increased intratumoral CD68^+^ staining. **(A)** Representative images showing overall CD68^+^ staining density in lung sections from iNOS WT (top) versus KO (bottom) mice in the survival cohort. **(B)** Quantification of overall tumor CD68^+^ staining, **(C)** representative images and **(D)** quantification of peritumoral CD68^+^ staining, and **(E)** representative images and **(F)** quantification of intratumoral CD68^+^ staining in iNOS WT (N = 5) versus KO mice (N = 5). NS = not significant. Mann–Whitney U test was used to compare overall, peritumoral, and intratumoral CD68^+^ between mice groups.

The role of iNOS in modulating the tumor immune microenvironment, particularly macrophages, is well established (Shikanai et al., 2023; Deniz et al., 2024; Liu X. et al., 2024). To explore the absence of iNOS on the number of macrophages, immunohistochemistry was performed on lung samples from five iNOS WT versus iNOS KO mice with a CD68 antibody, a pan-marker for both M1 and M2 macrophages. A pathologist blinded to sample identity scored CD68 staining patterns, including overall, peritumoral, and intratumoral intensity. CD68 staining in iNOS KO lung tissue exhibited non-statistical trend towards a higher overall intensity compared to the iNOS WT counterparts (Figures 4A,B), similar peritumoral staining (Figures 4C,D), and significantly higher intratumoral intensity (Figures 4E,F; t-test, P = 0.03). Thus, loss of iNOS is associated with elevated intratumoral CD68 staining.

## Discussion

In this study, we investigated the role of iNOS in a GEMM of LUAD driven by oncogenic *Kras* ^G12D^ and *Pten* loss. We find that iNOS loss does not alter the number of lung lesions, suggesting a minimal impact on tumor initiation in the absence of *Pten,* but increased tumor burden and reduced overall survival, pointing towards a tumor-suppressive role for iNOS in the context of *Pten* loss during tumor progression.

Loss of iNOS was associated with an increase in intratumoral CD68 staining, consistent with infiltration of lung tumors with macrophages. Mechanistically, iNOS-derived NO plays diverse roles in modulating immune responses, including regulation of tumor-associated macrophages, facilitation of T-cell infiltration, and maintenance of dendritic cell function (Somasundaram et al., 2020). These immunomodulatory effects may contribute to tumor suppression by enhancing anti-tumor immunity (Ridnour et al., 2015), or alternatively, tumor progression by promoting tumor growth in specific contexts (Gao et al., 2019). Perhaps related, it was reported that approximately half of human NSCLC specimens stained for iNOS exhibited dense infiltration of TILs positive for iNOS staining. Further, this pattern was associated with high PD-L1 and low HIF1α expression and correlated with improved overall survival, supporting a role for iNOS in anti-tumor immune responses and highlighting its potential as a biomarker of immune activation in NSCLC (Giatromanolaki et al., 2020). Similarly, high iNOS mRNA levels were reported to be more frequently in lower-grade NSCLC patient samples, and that high iNOS expression correlated with improved survival (Puhakka et al., 2003). Nevertheless, the finding that iNOS loss led to both an increase in intratumoral CD68 staining and reduced survival is not proof of a direct connection, and hence it remains to be determined the relationship of this correlation.

This study has several limitations, most notably the absence of detailed stromal profiling (e.g., cancer-associated fibroblast markers such as α-SMA or FAP) and lymphocytic characterization (e.g., CD3, CD8, B220) within the tumor microenvironment. Future inclusion of these markers will provided additional insight into whether the effects of iNOS loss extend beyond macrophage-driven changes to involve other stromal or immune components. Second, macrophage infiltration was assessed using CD68, a pan-macrophage marker that does not distinguish between pro-tumorigenic and anti-tumorigenic macrophage subsets. As a result, it is not possible to resolve whether the increased macrophage population in iNOS-deficient tumors is predominantly M1-like, M2-like, or functionally heterogeneous. Future work will address this gap by incorporating high-dimensional immune profiling, spatial transcriptomics, and single-cell analyses of lung tumors from these models to elucidate the broader mechanistic role of iNOS in LUAD progression. Third, we acknowledge that the limited sample size in the survival cohort resulted in a wide confidence interval for the hazard ratio. However, despite this limitation, the directional consistency between increased tumor burden and reduced survival in iNOS-deficient mice supports a role for iNOS loss in disease progression. Finally, reversal of iNOS loss from a tumor suppressive to tumor promoting role upon Pten loss remains to be eludicated, but again highlights the influence of context.

The potential of NOS inhibitors as cancer therapeutics has gained increasing attention due to the diverse roles of NO signaling in tumor progression, angiogenesis, and immune modulation (Lampson et al., 2012; Ridnour et al., 2015; Kruglyakov et al., 2023). However, this will undoubtably be context dependent. For example, the nonselective NOS inhibitor L-NAME was previously reported to suppress tumor growth and enhanced the therapeutic efficacy of carboplatin in a a *Kras* ^LSL−G12D^;*Trp53* ^−/−^ mouse model of NSCLC, suggesting a tumor-promoting role for NOS in lung cancer (Pershing et al., 2016). Similarly, iNOS loss in an Ad-Cre driven *Kras* ^*LSL-G12D*^ mouse model delayed lung tumorigenesis led to decrease number of lung lesions (Okayama et al., 2013). Conversely, we demonstrate that the loss of iNOS in the setting of *Pten* loss accelerated tumorigenesis. Admittedly, the number of variables limits comparisons (a general NOS inhibitor versus genetic ablation of iNOS, with or without a tumor suppressor, or even different tumor suppressors), but these differences underscore the complexity of NO signaling in lung cancer and highlight the critical role of genetic background, for example, the *Pten* status as shown here, which is a critical determinant of whether iNOS restrains or promotes lung tumor progression.

## Data Availability

The raw data supporting the conclusions of this article will be made available by the authors, without undue reservation.
